# Supplementary material for: Detection of Serum IgG Specific for Brachyspira pilosicoli and “Brachyspira canis” in Dogs
Source: Vet Sci. 2024 Jul 3;11(7):302. doi: 10.3390/vetsci11070302 (PMC11281529; doi:10.3390/vetsci11070302)
Supplement: Supplementary file 1 [file vetsci-11-00302-s001.zip › vetsci-3048441-supplementary.pdf]

Supplementary Table S1. Overview of the presumptive or definitive diagnoses and/or presenting complaints of the dogs (n = 168) from which serum samples were retrospectively utilized in the enzyme-linked immunosorbent assays (ELISAs). Sera were tested in the first, second or both ELISAs as indicated. Sera with increased levels of antibodies against *B. pilosicoli* are coloured using the same colour code as in Figure 5.

Sample ID	ELISA	Diagnosis/presenting complaints	Chronic enteropathy	Acute Diarrhea
1	2	Epistaxis	-	-
2	1	Spinal fracture, herniation, polytrauma, bite injury	-	-
3	2	Infection of unknown origin	-	-
4	2	Squamous cell carcinoma	-	-
5	2	Prostatitis	-	-
6	1, 2	Ataxia, weakness, regenerative anaemia	-	-
7	1, 2	TH3-L3 myelopathy	-	-
8	1, 2	Pancreatitis	-	-
9	1, 2	Pyometra	-	-
9	1, 2	Ovarian cysts, presumptive diagnosis (PD): neoplasia	-	-
10	1, 2	Blood donation	-	-
11	1, 2	Tenesmus, perineal hernia	-	-
12	2	Vestibular syndrome	-	-
13	2	Chronic temporomandibular arthralgia	-	-
14	1, 2	Chronic nephropathy	-	-
15	2	Annual check-up	-	-
16	1, 2	Seizures	-	-
17	1, 2	Coprostasis after bone feeding	-	+

Sample ID	ELISA	Diagnosis/presenting complaints	Chronic enteropathy	Acute Diarrhea
18	2	Cervical spine infarct	-	-
19	2	Acute haemorrhagic diarrhoea syndrome	-	+
20	1, 2	Vaginal neoplasia	-	-
21	2	Oesophageal stricture	-	-
22	2	Pyometra	-	-
23	2	Subaortic stenosis	-	-
24	1, 2	Chronic diarrhoea, food-responsive enteropathy, acute exacerbation with haematochezia and vomiting	+	+
25	2	Uncharacterised weakness	-	-
26	2	Mediastinal mass		
27	1, 2	Acute enteritis	-	+
28	2	Acute haemorrhagic diarrhoea syndrome	-	+
29	2	Neoplasia	-	-
30	2	Nasopharyngeal stenosis	-	-
31	2	Protein-losing enteropathy	+	+
32	2	Discopathy	-	-
33	2	Acute haemorrhagic diarrhoea syndrome	+	+
34	1, 2	Paraparesis	-	-
35	2	Acute enteritis	-	+
36	2	Vaginitis	-	-
37	1	Dermal mass, PD: dermoid cyst	-	-

Sample ID	ELISA	Diagnosis/presenting complaints	Chronic enteropathy	Acute Diarrhea
38	2	Annual check-up	-	-
39	2	Mesothelioma	-	-
40	2	Annual check-up	-	-
41	2	Pneumonia	-	-
42	2	Gastric foreign body, gastritis	-	-
43	2	Intoxication	-	-
44	1, 2	Immune-mediated polyarthritis, anaemia, dermal lesions	-	-
45	1, 2	Dystocia	-	-
46	1, 2	Gastric dilation/volvulus	-	-
47	1	Gastric dilation/volvulus	-	-
48	2	Chronic cough	-	-
49	2	Dystocia	-	-
50	1, 2	Pyometra, septic abdomen	-	-
51	1, 2	Cough, PD: pulmonary neoplasia	-	-
52	2	Check-up	-	-
53	2	Lymphoma	-	-
54	2	Immun-mediated haemolytic anaemia	-	-
55	2	Fractures due to trauma (accident)	-	-
56	2	Paraplegia	-	-
57	2	Prostatitis	-	-

Sample ID	ELISA	Diagnosis/presenting complaints	Chronic enteropathy	Acute Diarrhea
58	1, 2	Multifocal inflammatory disease of the central and peripheral nervous system	-	-
59	1	Polytrauma (hit by car)	-	-
60	2	Pericardial effusion	-	-
61	1, 2	Multicentric lymphoma	-	-
62	1, 2	Discospondylitis	-	-
63	1, 2	Pain, weight loss, diarrhoea, PD: acute enteritis	-	+
64	1, 2	Mitral valve endocardiosis, compensated	-	-
65	1, 2	Extramedullary spinal compression	-	-
66	1, 2	Myelopathy	-	-
67	1, 2	Gastric dilation/volvulus	-	-
68	1	Theobromine intoxication	-	-
69	1, 2	Ruptured splenic neoplasia	-	-
70	1, 2	Mycotoxin intoxication	-	-
71	1, 2	Trauma of the thoracic and abdominal wall	-	-
72	1	Pyometra	-	-
73	1, 2	Food-responsive enteropathy, acute exacerbation: haematochezia, vomiting	+	+
74	2	Intrahepatic portosystemic shunt	-	-
75	1, 2	Gastric dilation/volvulus	-	-

Sample ID	ELISA	Diagnosis/presenting complaints	Chronic enteropathy	Acute Diarrhea
76	1	Suspected ruptured splenic neoplasia	-	-
77	1, 2	Intoxication	-	-
78	1	Pleural effusion, pulmonary mass	-	-
79	1, 2	Urinary signs, urolithiasis	-	-
80	1, 2	Myasthenia gravis	-	-
81	1, 2	Pain of unknown origin	-	-
82	1, 2	Haemorrhagic gastroenteritis	-	+
83	2	Bite injury	-	-
84	1, 2	Bilateral cryptorchism, metastatic neoplasia	-	-
85	1, 2	Gastric dilation/volvulus	-	-
86	1, 2	Blood donation	-	-
87	2	Anterior uveitis	-	-
88	1, 2	PD: degenerative myelopathy	-	-
89	1, 2	Metastatic neoplasia	-	-
90	1, 2	PD: autoimmune haemolytic anaemia	-	-
91	1, 2	Immune-mediated haemolytic anaemia	-	-
92	1, 2	Polytrauma, bite injury	-	-
93	1, 2	Hyperadrenocorticism	-	-
94	1, 2	Mandibular osteosarcoma	-	-
95	1	Urolithiasis	-	-
96	2	Splenic tumour	-	-

Sample ID	ELISA	Diagnosis/presenting complaints	Chronic enteropathy	Acute Diarrhea
97	1, 2	Intoxication/gastroenteritis, acute: vomiting and soft faeces	-	+
98	1, 2	Gastric and jejunal foreign body, dyschezia	-	-
99	2	Disseminated intravascular coagulopathy (paraneoplastic syndrome)	-	-
100	2	Mast cell tumour	-	-
101	2	Chronic rhinitis	-	-
102	2	Lymphoma	-	-
103	2	Nerve sheath tumour	-	-
104	2	Mandibular fracture	-	-
105	2	Prostatitis, cystitis	-	-
106	2	Discospondylitis	-	-
107	2	Idiopathic epilepsy	-	-
108	2	Lipoma	-	-
109	2	Urothelial carcinoma	-	-
110	2	Neoplasia	-	-
111	2	Idiopathic epilepsy	-	-
112	2	Lymphoma	-	-
113	1, 2	Discopathy C4-C5	-	-
114	2	Discopathy C6-C7	-	-
115	2	Peripheral neuropathy	-	-
116	2	Oral fibrosarcoma	-	-
117	2	Necrotising meningoencephalitis	-	-

Sample ID	ELISA	Diagnosis/presenting complaints	Chronic enteropathy	Acute Diarrhea
118	2	Urolithiasis, prostatomegaly	-	-
119	2	Uterine prolapse	-	-
120	2	Intestinal neoplasia	-	-
121	1, 2	Hepatic neoplasia, haematuria, ascites	-	-
122	2	Lymphoblastic leukaemia	-	-
123	1, 2	Hyphaema, anterior and posterior uveitis; enucleation	-	-
124	1, 2	Fibrocartilaginous embolism	-	-
125	1, 2	Multicentric lymphoma	-	-
126	1, 2	Pyometra	-	-
127	2	Acute haemorrhagic diarrhoea syndrome	-	+
128	1, 2	Splenic neoplasia	-	-
129	1, 2	Leukaemia	-	-
130	2	Cystitis	-	-
131	2	PD: intestinal neoplasia	-	-
132	2	Uncgaracterised weakness	-	-
133	1, 2	Geriatric vestibular syndrome	-	-
134	2	Corneal ulcer	-	-
135	2	Intoxication	-	-
136	1, 2	Discopathy TH13-L1	-	-
137	2	Oesophageal perforation	-	-
138	1, 2	Intoxication and PD: oesophageal and gastric ulceration	-	-

Sample ID	ELISA	Diagnosis/presenting complaints	Chronic enteropathy	Acute Diarrhea
139	2	Gastric dilation/volvulus	-	-
140	2	Dirofilariasis, pneumothorax	-	-
141	2	Lameness	-	-
142	1, 2	Splenic and hepatic neoplasia	-	-
143	1, 2	Gastric ulceration	-	-
144	2	Acute haemorrhagic diarrhoea syndrome	-	+
145	1, 2	Ischemic myelopathy	-	-
146	1, 2	Hit by car accident/sensitive stomach	-	-
147	1, 2	Splenic neoplasia	-	-
148	2	Pulmonary hypertension	-	-
149	1, 2	Laryngeal paralysis/rhinitis	-	-
150	1, 2	Acute renal failure due to leptospirosis	-	-
151	2	Oesophageal stricture	-	-
152	2	Leishmaniasis	-	-
153	2	Chronic enteropathy	+	+
154	2	Food-responsive enteropathy	+	-
155	2	Food-responsive enteropathy	+	-
156	2	Intoxication	-	-
157	2	Regenerative anaemia	-	-
158	1, 2	Gastric foreign body	-	-
159	2	Recurrent vomiting	+	-
160	2	Lymphoma	-	-
161	2	Intoxication	-	-

Sample ID	ELISA	Diagnosis/presenting complaints	Chronic enteropathy	Acute Diarrhea
162	2	Vomiting of unknown cause	-	-
163	2	Dyspnoea	-	-
164	1, 2	Carprofen intoxication	-	-
165	2	PD: chronic inflammatory enteropathy	+	+
166	2	Gastric ulceration, primary immune-mediated haemolytic anaemia	-	-
167	2	Elective surgery (neutering)	-	-
168	1	No pre-existing condition	-	-
