# Supplementary material for: Detection of Serum IgG Specific for Brachyspira pilosicoli and “Brachyspira canis” in Dogs
Source: Vet Sci. 2024 Jul 3;11(7):302. doi: 10.3390/vetsci11070302 (PMC11281529; doi:10.3390/vetsci11070302)

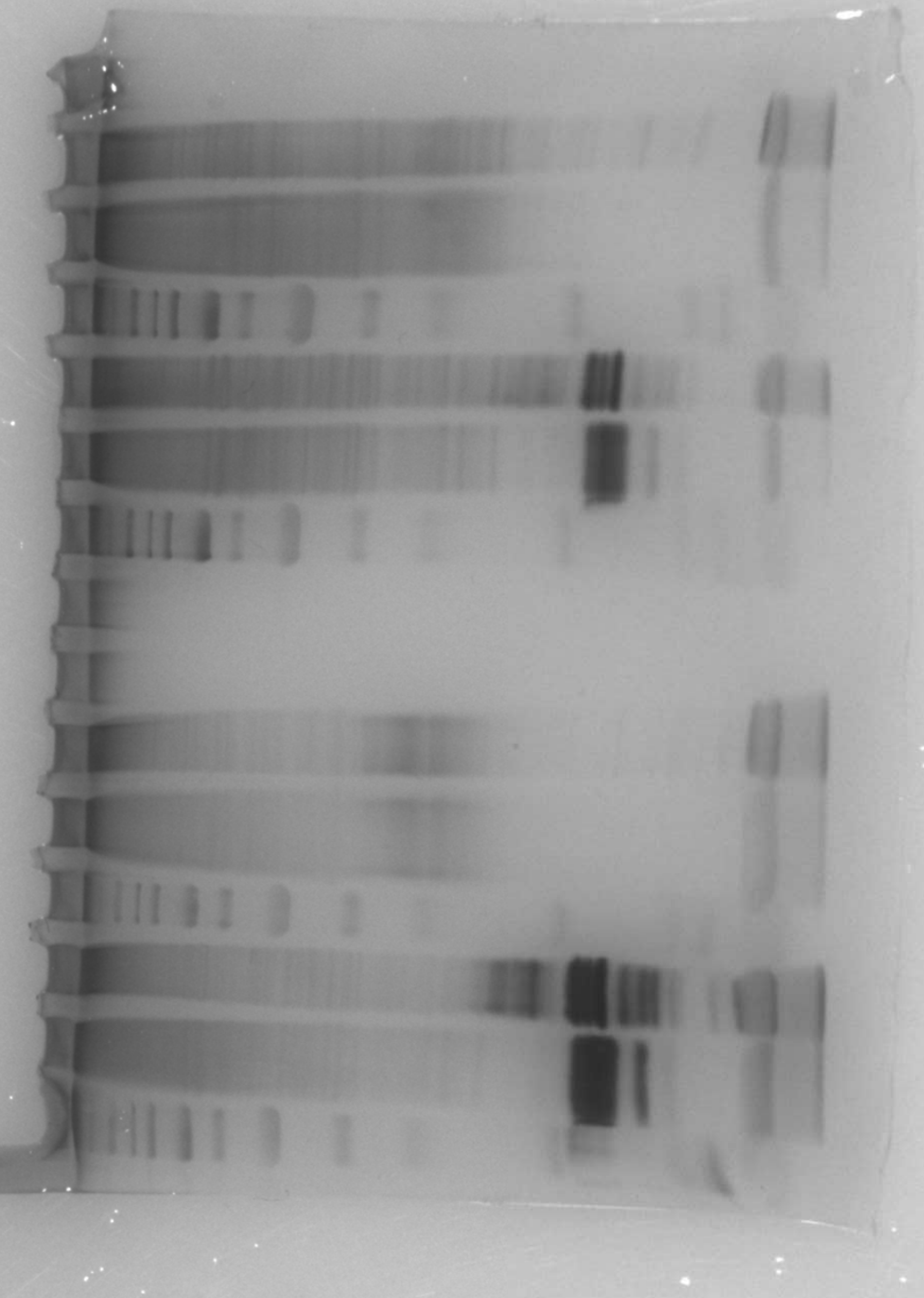

Supplement: Supplementary file 1 [file figure 6A.pdf › figure 6A.pdf]
